# CircASXL1 knockdown represses the progression of colorectal cancer by downregulating GRIK3 expression by sponging miR-1205

**DOI:** 10.1186/s12957-021-02275-6

**Published:** 2021-06-14

**Authors:** Guojiu Fang, Yibin Wu, Xueli Zhang

**Affiliations:** 1 Department of General Surgery, Shanghai Fengxian Central Hospital, No. 6600, Nanfeng Road, Nanqiao New Town, Fengxian District, Shanghai, 201400 China; 2 Department of Liver Surgery, Shanghai Cancer Center, Fudan University, Shanghai, 200032 China

**Keywords:** CRC, circASXL1, miR-1205, GRIK3, Guojiu Fang and Yibin Wu contribute equally.

## Abstract

**Background:**

Colorectal cancer (CRC) is a common aggressive tumor that poses a heavy burden to human health. An increasing number of studies have reported that circular RNA (circRNA) is involved in the progression of CRC. In this study, the special profiles of circASXL1 (circ_0001136) in CRC progression were revealed.

**Methods:**

The expression of circASXL1, microRNA-1205 (miR-1205), and glutamate ionotropic receptor kainate type subunit 3 (GRIK3) mRNA was detected by quantitative real-time polymerase chain reaction (qRT-PCR). The protein expression was determined by Western blot or immunohistochemistry. Cell colony-forming ability was investigated by colony formation assay. Cell cycle and apoptosis were demonstrated using cell-cycle and cell-apoptosis analysis assays, respectively. Cell migration and invasion were detected by wound-healing and transwell migration and invasion assays, respectively. The binding sites between miR-1205 and circASXL1 or GRIK3 were predicted by circBank or miRDB online database, and identified by dual-luciferase reporter assay. The impact of circASXL1 on tumor formation in vivo was investigated by in vivo tumor formation assay.

**Results:**

CircASXL1 and GRIK3 expression were apparently upregulated, and miR-1205 expression was downregulated in CRC tissues and cells relative to control groups. CircASXL1 knockdown inhibited cell colony-forming ability, migration and invasion, whereas induced cell arrest at G0/G1 phase and cell apoptosis in CRC cells; however, these effects were attenuated by miR-1205 inhibitor. Additionally, circASXL1 acted as a sponge for miR-1205, and miR-1205 was associated with GRIK3. Furthermore, circASXL1 silencing hindered tumor formation by upregulating miR-1205 and downregulating GRIK3 expression.

**Conclusion:**

CircASXL1 acted an oncogenic role in CRC malignant progression via inducing GRIK3 through sponging miR-1205. Our findings provide a theoretical basis for studying circASXL1-directed therapy for CRC.

**Supplementary Information:**

The online version contains supplementary material available at 10.1186/s12957-021-02275-6.

## Background

Colorectal cancer (CRC) is a pernicious colonic or rectal tumor, which is one of the main causes of cancer-correlated mortalities worldwide [1–3]. Although much progress has been achieved in CRC therapy or prevention strategies [4–6], the 5-year overall survival rate of CRC cases is only about 10% owing to the high recurrence rate and distant metastatic ability [7]. Therefore, further studying of the molecular mechanism behind CRC development is necessary to develop novel therapeutic targets for CRC to improve the poor clinical outcome.

Circular RNA (circRNA) is a novel noncoding RNA and is formed by back-splicing events as a covalently closed loop, and thereby is more stable than linear RNA [8, 9]. The dysregulation of circRNA has been revealed to be correlated with cancer progression, which implies that circRNA has a potential role in cancer progression [10]. Previous studies have explained that circRNA participates in the regulation of CRC process. For instance, circ_004680 facilitated CRC cell proliferation and migration [11]; circ_0007142 silencing hindered cell proliferation and tumor metastasis by sponging microRNA-122-5p (miR-122-5p) in CRC [12]. In another example, Tian et al. explained that circ_0025033 accelerated CRC cell proliferation and invasion, whereas inhibited cell apoptosis via binding to miR-143-3p [13]. ASXL transcriptional regulator 1 (ASXL1), a cancer-associated gene, has been reported to hinder cell growth in CRC [14]; however, whether circASXL1, a circRNA formed from ASXL1 gene, participates in CRC progression remains unknown. Thus, this study was designed to reveal the role of circASXL1 in CRC progression.

MiRNAs are a group of small noncoding RNAs with about 20 nucleotides and mainly control gene expression by interacting with their 3′-untranslated regions (3′UTR) [15]. Recent reports have indicated that miRNA is involved in the pathogenesis of various diseases, including cancers [16, 17]. Accumulating evidences showed that miRNA acted as a tumor suppressor or promoter in CRC [18, 19]. In particular, miR-1205 was indicated to inhibit the progression of lung cancer [20], ovarian cancer [21], and gastric cancer [22]. However, the role of miR-1205 in CRC process remains to be explored.

Glutamate ionotropic receptor kainate type subunit 3 (GRIK3) belongs to glutamate kainate receptor family, and exerts vital roles in neuroactive ligand receptor interaction pathway [23]. Existing evidences showed that GRIK3 participated in cancer progression. For example, Xiao et al. unveiled that GRIK3 contributed to epithelial-mesenchymal transition (EMT) in breast cancer [24]. Gong et al. reported that GRIK3 was a tumor promoter and was correlated with lymph node metastasis in gastric cancer [23]. However, the effects and mechanism of GRIK3 in CRC development are still unclear.

Herein, the expression of circASXL1, miR-1205, and GRIK3 in CRC tissues and cells was determined. The function and regulatory mechanism of circASXL1 in CRC malignant progression were revealed.

## Materials and methods

### Tissue acquirement and storage

Twenty-three pairs of human CRC tissues and paracancerous normal intestinal tissues were collected from CRC patients from Shanghai Fengxian Central Hospital. Total tissues were stored at − 80 °C in a freezer. This study was approved by the Ethics Committee of the Shanghai Fengxian Central Hospital and the CRC patient related to this experiment signed the written informed consent.

### Cell culture

Human CRC cell lines (SW480 and SW620) and human normal intestinal epithelial cell line NCM460 were purchased from Otwo Biotech (Shenzhen, China). Cells were cultivated in Dulbecco’s modified Eagle’s medium (DMEM; Biosun, Shanghai, China) supplemented with 10% fetal bovine serum (FBS; Biosun) and 1% streptomycin/penicillin (Millipore, Bradford, MA, USA) at 37 °C in an incubator with 5% CO_2_.

### Cell transfection

The small interfering RNAs targeting circASXL1 (si-circASXL1 and si-circASXL1#2), the mimic of miR-1205 (miR-1205 mimic; a chemically synthesized double-stranded mature sequence), the overexpression plasmid of circASXL1 (oe-circASXL1), the inhibitor of miR-1205 (miR-1205 inhibitor; a chemically synthesized double-stranded sequence), the overexpression vector of GRIK3 (pcDNA-GRIK3), the small hairpin RNA against circASXL1 (sh-circASXL1), and respective controls (si-NC, miR-NC mimic, oe-NC, miR-NC inhibitor, pcDNA-NC and sh-NC) were built by Invitrogen Co., Ltd. (Carlsbad, CA, USA). Constructed plasmids or oligonucleotides were transfected into cells using Lipofectamine 2000 (Invitrogen) according to the published methods [25]. The sequences in this part were displayed in Table S1.

### Quantitative real-time polymerase chain reaction

CRC tissues and cells were lysed with TransZol (TransGen, Beijing, China), and total RNA was extracted using an RNAsimple kit (Tiangen, Beijing, China). After performing concentration measure, RNA was reversely transcribed into cDNA using a FastKing RT Kit (Tiangen) or MiX-x™ synthesis Kit (TaKaRa, Dalian, China). Following that, SuperReal PreMix Color (Tiangen) was performed to detect the expression levels of circASXL1, miR-1205, or GRIK3. Data were assessed with the 2^-∆∆Ct^ method with U6 and glyceraldehyde 3-phosphate dehydrogenase (GAPDH) as references. The sequences of forward (F) and reverse (R) primers were listed in Table S1.

### RNase R treatment assay

RNase R treatment assay was performed based on the published reference [26]. In brief, SW480 and SW620 cells were harvested and treated with TransZol (TransGen). And RNA was extracted using an RNAsimple kit (Tiangen). Following that, 1 μg RNA was incubated with 3 U of RNase R (Epicentre, Madison, WI, USA) at 37 °C for 30 min. Then, RNeasy MinElute Cleaning Kit (QIAGEN, Valencia, CA, USA) was carried out to purify RNA. The amount of circASXL1 and ASXL1 mRNA was detected by quantitative real-time polymerase chain reaction (qRT-PCR) with ASXL1 mRNA as a reference.

### Colony formation assay

This assay was carried out as previously reported [27]. Briefly, SW480 and SW620 cells were grown in 6-well plate (500 cells per well) for 16 h. After various treatments, cells were cultured for another 2 weeks. And DMEM (Biosun) was replaced every 3 days. The positive colonies were immobilized and stained using paraformaldehyde (Beyotime, Jiangsu, China) and crystal violet (Beyotime), respectively. Cell colony-forming ability was assessed by counting the number of colonies manually. A colony was defined when the number of cells more than 50.

### Cell-cycle analysis by flow cytometry

Cell cycle detection kit (Yeasen, Shanghai, China) was employed in this part according to the manufacturer’s instruction. In short, SW480 and SW620 cells were collected after digested using trypsin (Thermo Fisher, Waltham, MA, USA). And cells were washed twice with phosphate buffer solution (PBS; Thermo Fisher). Then, anhydrous ethanol (Millipore) was used to fix the cells overnight at 4 °C, and then incubated with RNase A (Millipore) at 37 °C for 30 min in water, followed by the incubation with 500 μL propidium iodide (PI; Yeasen) at room temperature for 30 min. Samples were assessed by a flow cytometry (BD Biosciences, San Diego, CA, USA).

### Cell-apoptosis analysis by flow cytometry

This assay was employed to detect cell apoptosis with Annexin V-fluorescein isothiocyanate (Annexin V-FITC)/PI apoptosis detection kit (Yeasen) as per guidebook. Briefly, the transfected cells were harvested and then suspended in 100 μL binding buffer (Yeasen), followed by the incubation with Annexin V-FITC (Yeasen) and PI (Yeasen) at room temperature for 12 min in dark. Cell apoptotic rate was revealed after samples were assessed with a flow cytometry (BD Biosciences).

### Western blot

CRC tissues and cells were treated with lysis buffer (Beyotime) to prepare protein samples. Protein concentration was measured using bicinchoninic acid assay (BCA) kit (Beyotime). And 20 μg protein was loaded on 12% bis-tris-acrylamide gel (Thermo Fisher). Then, protein bands were transferred onto polyvinylidene fluoride (Millipore) and immersed in 5% nonfat milk (Solarbio, Beijing, China) at 4 °C for 4 h. After incubated with anti-B cell lymphoma-2 (anti-Bcl-2) (1:1000; CST, Boston, MA, USA), anti-BCL2-associated x protein (anti-Bax) (1:1000; CST), anti-GRIK3 (1:1000; Abcam, Cambridge, UK), and anti-β-Actin (1:1000; CST) at 4 °C overnight, the membranes were incubated with the secondary antibody labeled with horseradish peroxidase (1:2000; CST) at 37 °C for 2 h. The protein bands were developed using a Clarity™ ECL Substrate Kit (Bio-Rad, Shanghai, China). β-Actin was chosen as a control.

### Wound-healing assay

Wound-healing assay was conducted according to standard protocols [28]. In brief, SW480 and SW620 cells were seeded in 6-well plate for 16 h, and constructed plasmids or oligonucleotides were transfected into the cells. Wounds were created using 10-μL pipette tips when the confluence of cells reached about 100%. Following that, floated cells and debris were discarded with PBS (Thermo Fisher). Then, serum-free DMEM (Biosun) was placed into the wells for 24 h. The wound width was determined by calculating the area occupied by migrated cells under a microscope (Olympus) with × 40 magnification. To exclude cell proliferation-caused effect on cell migration, the wound-healing assay was performed in a similar approach as shown above expect that each well was incubated with mitomycin C (Sigma, St. Louis, MO, USA).

### Transwell migration and invasion assays

The migration and invasion of SW480 and SW620 cells were assessed using transwell chamber without or with Matrigel (Corning, New York, Madison, USA), respectively, as previously described [29]. Shortly, cells were suspended in serum-free DMEM (Biosun), and added into the upper chamber. DMEM containing 15% FBS (Biosun) was placed into the lower chamber. The cells were cultured for 24 h and medium was discarded. Methanol (Beyotime) and crystal violet (Beyotime) were severally employed to fix and dye cells. The migrated or invaded cells were analyzed under a microscope (Olympus) with × 100 magnification.

### Dual-luciferase reporter assay

The targeting sites between miR-1205 and circASXL1 or GRIK3 3′UTR were assessed using circBank or miRDB online database. Subsequently, the wild-type (WT) plasmids of circASXL1 and the 3′UTR of GRIK3 (WT-circASXL1 and WT-GRIK3 3’UTR) were built by inserting the complete sequence of circASXL1 and 150 base pairs of GRIK3 3′UTR into GV272 (GeneChem, Shanghai, China). And the mutant plasmids of circASXL1 and GRIK3 (MUT-circASXL1 and MUT-GRIK3 3′UTR) were established in the same manner. Constructed plasmids were transfected into SW480 and SW620 cells using Lipofectamine 2000 (Invitrogen) according to the published reference [29]. The binding relationship was demonstrated by detecting luciferase activity using a Dual-Lucy Assay Kit (Solarbio) with *Ranilla* Luciferase activity as a reference.

### In vivo tumor formation assay

Charles River (Beijing, China) provided the male BALB/c nude mice (5-week-old). Nude mice were fed in pathogen-free condition. All mice were divided into the 2 groups: sh-NC group and sh-circASXL1 group (*N* = 5, respectively). 2 × 10^6^ SW480 cells transfected with sh-circASXL1 or sh-NC were diluted using PBS (Thermo Fisher) and subcutaneously inoculated into the mice. After 7 days, tumor volume was measured every 1 week. On the 35th day, all mice were killed and tumors were excised. The volume and weight of tumors and the expression of circASXL1, miR-1205 and GRIK3 were assessed.

### Immunohistochemistry analysis

The neoplasms from in vivo tumor formation assay were cut into 4-μm-thick sections. Then, the immunohistochemistry (IHC) analysis was carried out according to the published methods [30]. In brief, the sections were fixed, dehydrated, and embedded. The sections immersed in sodium citrate were heated for antigen retrieval. Then, the tissues were incubated with anti-GRIK3 (1:100; Cusabio Biotech, Wuhan, China), anti-nuclear proliferation marker (anti-Ki67) (1:100; Affinity, Nanjing, China) and secondary antibody (1:200; Affinity), respectively. The staining results were assessed with a microscope (Leica Microsystems, Mannheim, Germany). Semiquantitative assessment was performed based on the percentage of dyed cells and intensity of immunostaining according to the published methods [31].

### Statistical analysis

Data were assessed using SPSS 21.0 software (IBM, Somers, NY, USA) based on at least 3 replicates. Data were presented as means ± standard deviations (SD). Significant differences were compared with two-tailed Student’s *t* tests between the two groups, and analyzed with Wilcoxon rank-sum test or one-way analysis of variance among three or more groups. *P* value < 0.05 was considered statistically significant.

## Results

### CircASXL1 expression was obviously upregulated in CRC tissues and cells

**Fig. 1:**
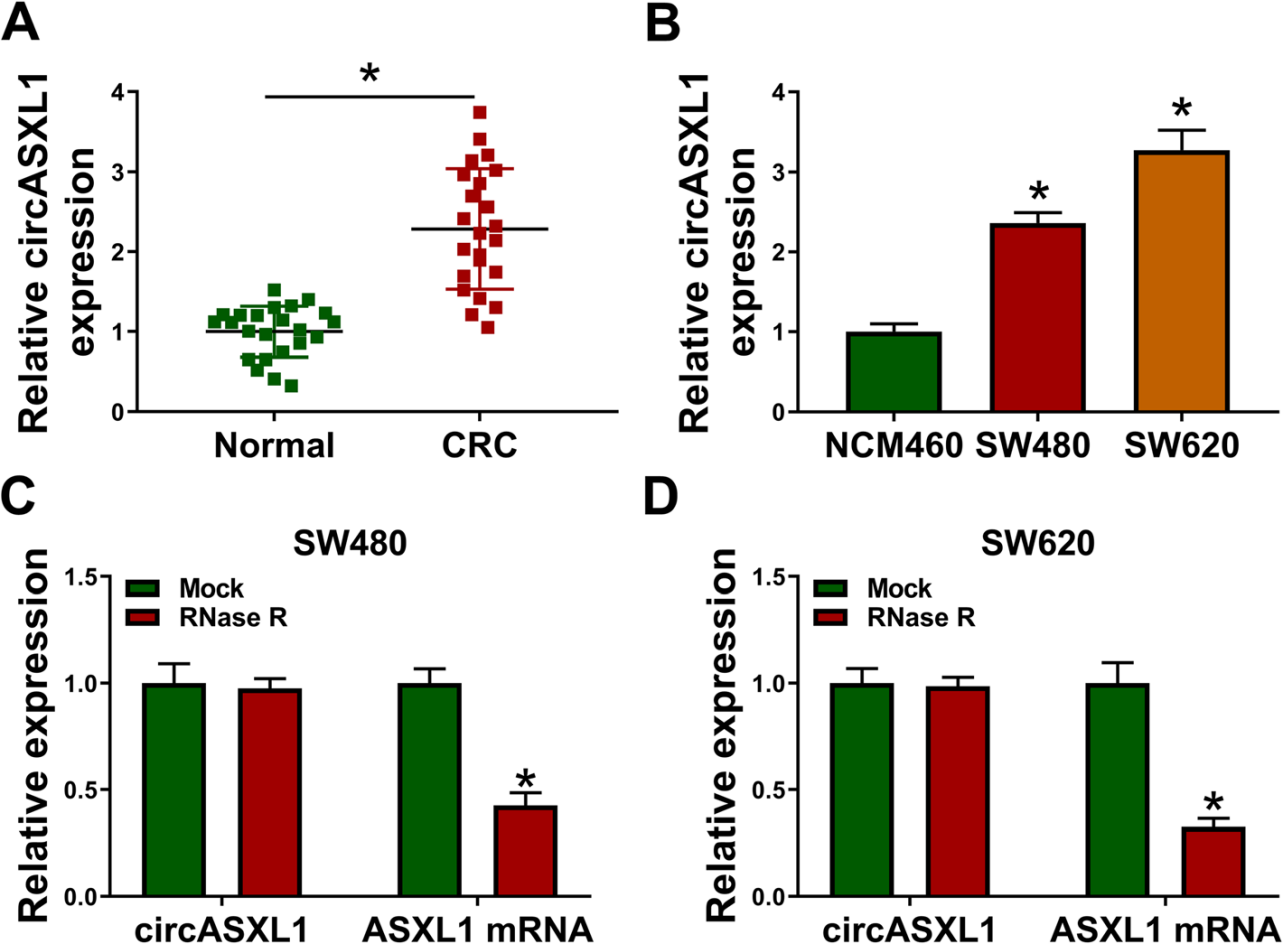
CircASXL1 was highly expressed in CRC tissues and cells. **A**, **B** CircASXL1 expression was increased in CRC tissues as well as SW480 and SW620 cells. **C**, **D** RNase R treatment assay showed circASXL1 was more stable than linear ASXL1 in SW480 and SW620 cells. The comparison in (**A**) was assessed with Wilcoxon rank-sum test, in (**B**) with ANOVA and in (**C** and **D**) with two-tailed Student’s *t* tests. *P < 0.05

To figure out the expression pattern of circASXL1, located in chr20:30954186-30956926 and formed from exons 2 and 3 of ASXL1 (Fig. S1), in CRC tissues, we firstly predicted circASXL1 expression through GEO dataset (GSE142837). Results showed that circASXL1 was highly expressed in CRC tissues when compared with that in normal intestinal tissues (Fig. S2A). Also, the data from qRT-PCR showed that circASXL1 expression was obviously increased in CRC tissues and SW480 and SW620 cells in comparison with that in normal intestinal tissues and NCM460 cells, respectively (Fig. 1A, B). The overall survival curve of CRC patients with high or low circASXL1 expression was further assessed. As shown in Fig. S2B, the CRC sufferers with high circASXL1 expression had poor prognosis when compared with these with low circASXL1 expression. Meanwhile, it was found using RNase R treatment assay that circASXL1 was more stable than linear ASXL1 (Fig. 1C, D). These data indicated that the high expression of circASXL1 might be associated with CRC progression.

### CircASXL1 knockdown repressed cell proliferation, migration and invasion, whereas induced cell arrest in G0/G1 phase and cell apoptosis in CRC

**Fig. 2:**
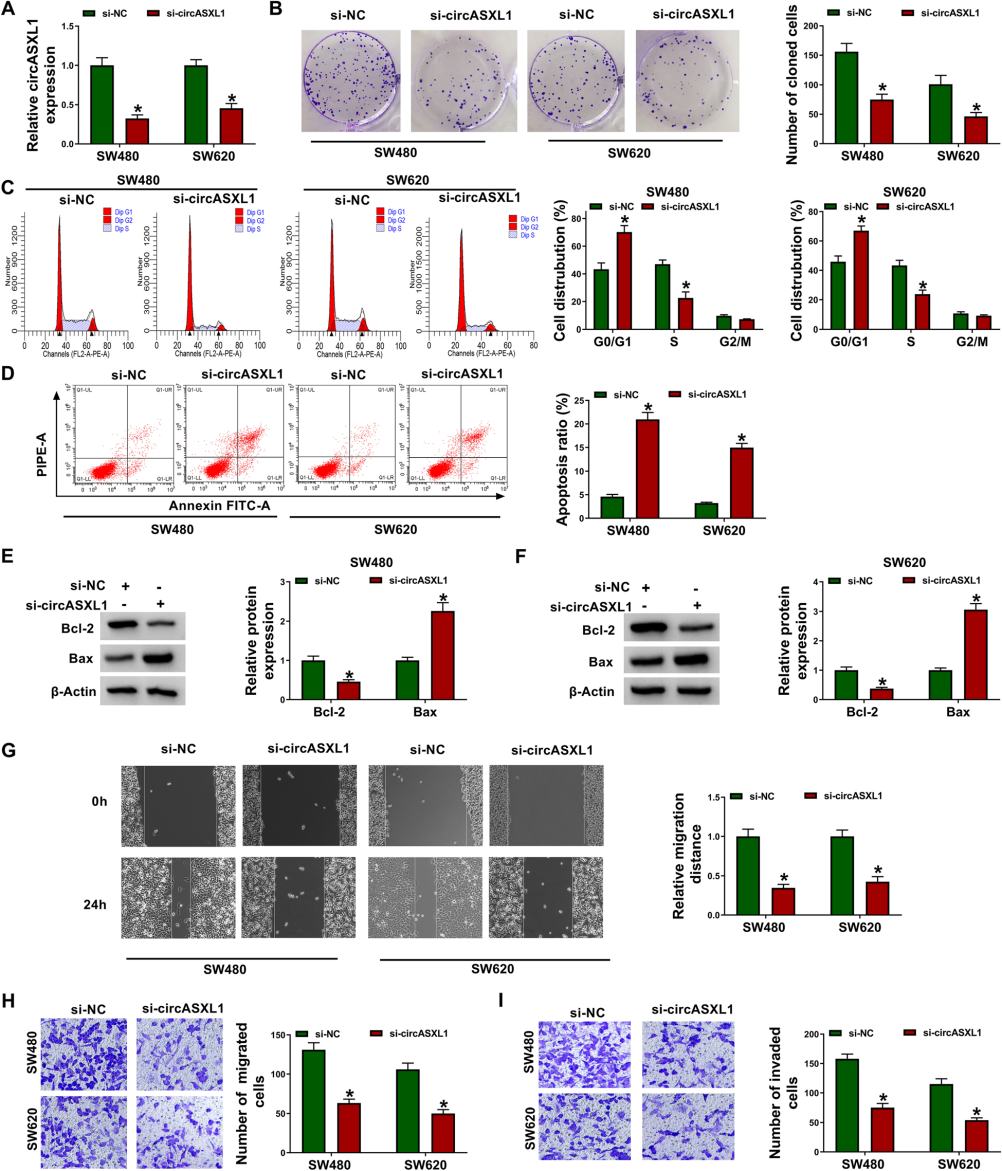
CircASXL1 silencing suppressed CRC progression. **A** CircASXL1 silencing reduced circASXL1 expression in SW480 and SW620 cells. **B** CircASXL1 knockdown repressed colony-forming ability of SW480 and SW620 cells. **C**, **D** CircASXL1 silencing induced cell arrest in G0/G1 phase and cell apoptosis in SW480 and SW620 cells. **E**, **F** CircASXL1 silencing downregulated Bcl-2 protein expression, and upregulated Bax protein expression. **G**–**I** CircASXL1 absence inhibited the migration and invasion of SW480 and SW620 cells. Significant differences were compared with two-tailed Student’s *t* tests. *P < 0.05

To reveal that whether circASXL1 participated in the regulation of CRC progression, we investigated the effects of circASXL1 knockdown on cell proliferation, cell cycle, migration, invasion, and apoptosis in SW480 and SW620 cells. The siRNAs of circASXL1 were firstly built, and their high efficiency in downregulating circASXL1 was shown in Fig. 2A and Fig. S3A. Subsequently, it was found that circASXL1 silencing inhibited cell colony-forming ability and induced cell arrest in G0/G1 phase in SW480 and SW620 cells (Fig. 2B, C, Fig. S3B and S3C), suggesting that circASXL1 knockdown repressed cell proliferation. Additionally, the apoptosis of SW480 and SW620 cells was induced after circASXL1 silencing (Fig. 2D and Fig. S3D). To evidently explain the effect of circASXL1 on cell apoptosis, the expression of apoptosis-related proteins (Bcl-2 and Bax) was detected by western blot in the SW480 and SW620 cells transfected with si-circASXL1 or si-NC. As a result, we found that circASXL1 knockdown strikingly downregulated Bcl-2 protein expression and upregulated Bax protein expression (Fig. 2E, F, Fig. S3E, and S3F), which meant circASXL1 depletion repressed cell apoptosis. Furthermore, it was found that circASXL1 knockdown inhibited the migration and invasion of SW480 and SW620 cells (Fig. 2G–I and Fig. S3G–I). To exclude cell proliferation-caused effect on cell migration, mitomycin C was employed in wound-healing assay. As shown in Fig. S4A and B, circASXL1 depletion also repressed cell migration under treatment of mitomycin C. Thus, the above data demonstrated that circASXL1 might act as a tumor promoter in CRC progression.

### CircASXL1 functioned as a sponge of miR-1205 in CRC cells

**Fig. 3:**
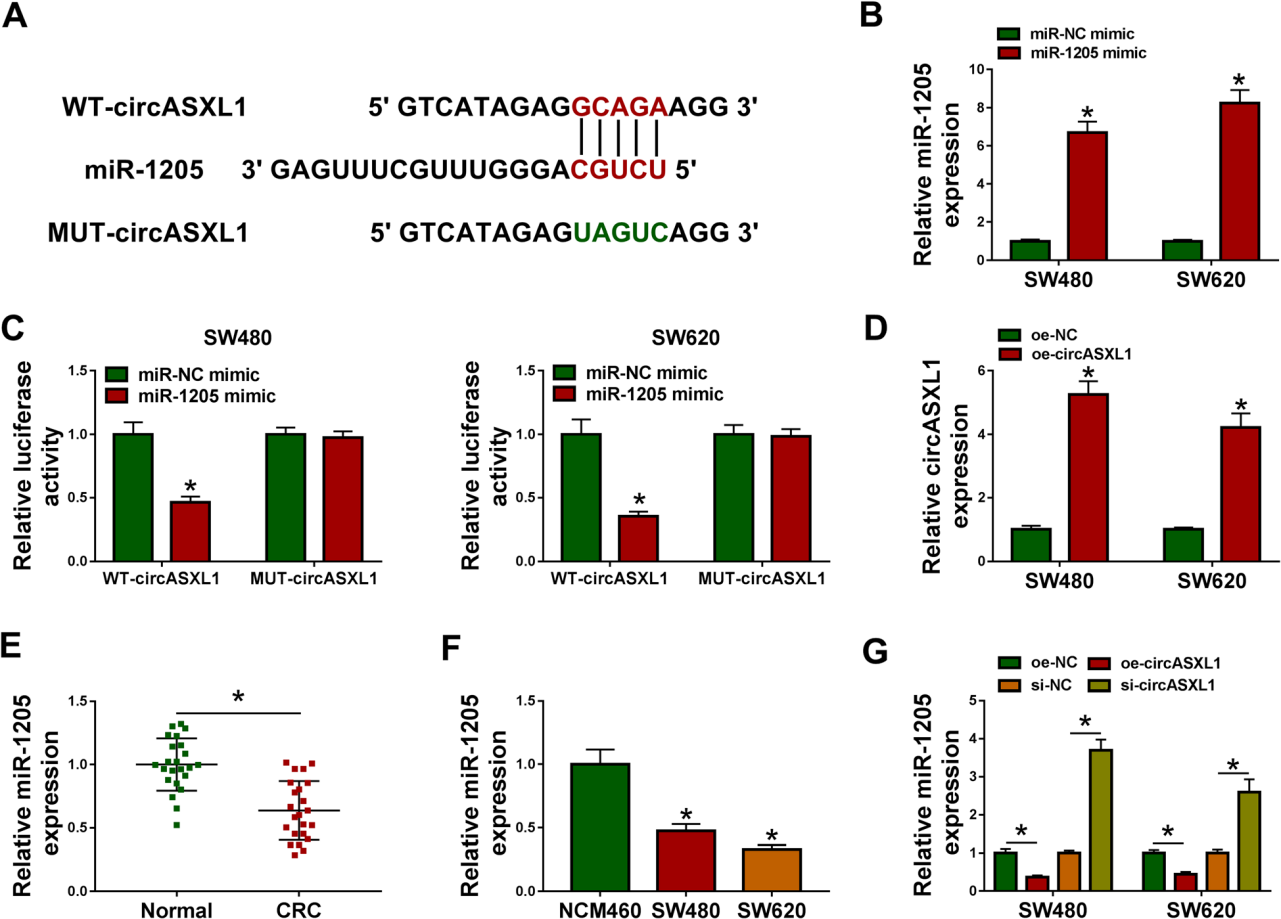
CircASXL1 was associated with miR-1205 in CRC cells. **A** CircBank online database was employed to predict the binding sites between circASXL1 and miR-1205. **B**, **D** The high overexpression efficiency of miR-1205 mimic and oe-circASXL1 was determined. **C** The interaction between circASXL1 and miR-1205 was identified by dual-luciferase reporter assay. **E**, **F** MiR-1205 was weakly expressed in CRC tissues as well as SW480 and SW620 cells. **G** CircASXL1 overexpression decreased miR-1205 expression, and circASXL1 knockdown increased miR-1205 expression. Significant differences in (**B**–**D**) were compared with two-tailed Student’s *t* tests, in (**E**) with Wilcoxon rank-sum test and in (**F** and **G**) with ANOVA. *P < 0.05

To unveil the underneath mechanism by which circASXL1 regulated CRC progression, we predicted the circASXL1-associated miRNA(s) by circBank online database. Results showed that there were 6 miRNAs containing more binding sites of circASXL1, including miR-1-3p, miR-1205, miR-206, miR-34b-5p, miR-616-3p, and miR-767-3p. It was analysis using qRT-PCR that miR-1205 expression was the highest in the circASXL1-knockdowned cells when compared with control groups (Fig. S5). Thus, miR-1205 was employed as a follow-up subject. The binding sequence between circASXL1 and miR-1205 as well as the mutant sites of circASXL1 were also presented in Fig. 3A. Subsequently, to confirm the interaction between circASXL1 and miR-1205, the overexpression efficiency of miR-1205 mimic and oe-circASXL1 was detected. As expected, miR-1205 mimic dramatically increased miR-1205 expression, and oe-circASXL1 obviously upregulated circASXL1 expression in SW480 and SW620 cells compared with control groups (Fig. 3B, D), suggesting the high efficiency of miR-1205 mimic and oe-circASXL1 in increasing miR-1205 or oe-circASXL1 expression. Additionally, dual-luciferase reporter assay presented that the luciferase activity of WT-circASXL1 and miR-1205 mimic group was apparently repressed, whereas the luciferase activity had no prominent change in co-transfection group of MUT-circASXL1 and miR-1205 mimic (Fig. 3C), which confirmed that circASXL1 could bind to miR-1205 by the complementary sites between them. The expression of miR-1205 in CRC tissues and cells were continued to be detected. QRT-PCR results showed that miR-1205 expression was apparently downregulated in CRC tissues as well as SW480 and SW620 cells as compared to control groups (Fig. 3E, F). Furthermore, it was found that circASXL1 overexpression significantly repressed miR-1205 expression and circASXL1 knockdown prominently increased miR-1205 expression (Fig. 3G), showing that circASXL1 negatively regulated miR-1205 in CRC cells. These evidences manifested that circASXL1 was associated with miR-1205 in SW480 and SW620 cells.

### CircASXL1 silencing hindered CRC progression via sponging miR-1205

**Fig. 4:**
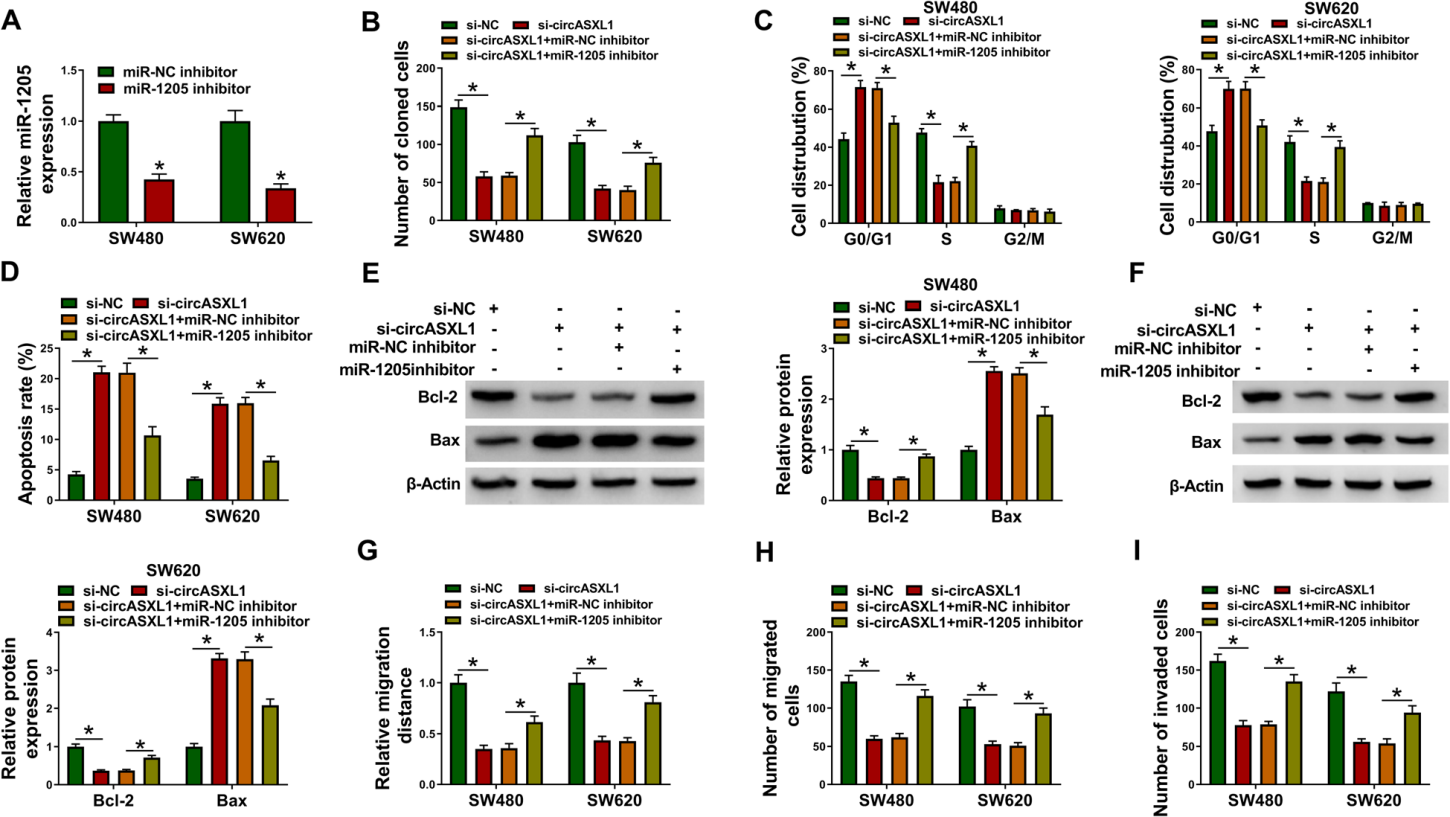
CircASXL1 modulated CRC development by associating with miR-1205. **A** The high silencing efficiency of miR-1205 inhibitor was determined by qRT-PCR. **B** MiR-1205 inhibitor attenuated circASXL1 knockdown-mediated effects on cell colony-forming ability. **C**, **D** The effects of circASXL1 knockdown on cell cycle process and cell apoptosis were impaired by miR-1205 repression. **E**, **F** The impacts of circASXL1 silencing on the protein expression of Bcl-2 and Bax were reversed after miR-1205 repression. **G**–**I** The inhibitory effects of circASXL1 absence on the migration and invasion of SW480 and SW620 cells were restrained by miR-1205 inhibitor. Significant differences in (**A**) were compared with two-tailed Student’s *t* tests and in (**B**–**I**) with ANOVA.*P < 0.05

Given the interaction between circASXL1 and miR-1205, this study continued to demonstrate that whether miR-1205 participated in circASXL1-mediated CRC development. QRT-PCR results firstly presented that miR-1205 inhibitor was effective in reducing miR-1205 expression (Fig. 4A). Then, it was found that miR-1205 inhibitor attenuated the inhibition of circASXL1 knockdown on cell colony-forming ability and the promotion of that on cell cycle arrest (Fig. 4B, C). MiR-1205 inhibitor restored circASXL1 silencing-induced apoptosis of SW480 and SW620 cells (Fig. 4D). Meanwhile, results showed that circASXL1 silencing visibly downregulated Bcl-2 protein expression and upregulated Bax protein expression in SW480 and SW620 cells, whereas these effects were restrained by miR-1205 inhibitor (Fig. 4E, F). Furthermore, miR-1205 inhibitor hindered the inhibitory influences of circASXL1 knockdown on the migration and invasion of SW480 and SW620 cells (Fig. 4G–I). The above data demonstrated that circASXL1 regulated CRC process via binding to miR-1205.

### MiR-1205 was associated with GRIK3 in CRC cells

**Fig. 5:**
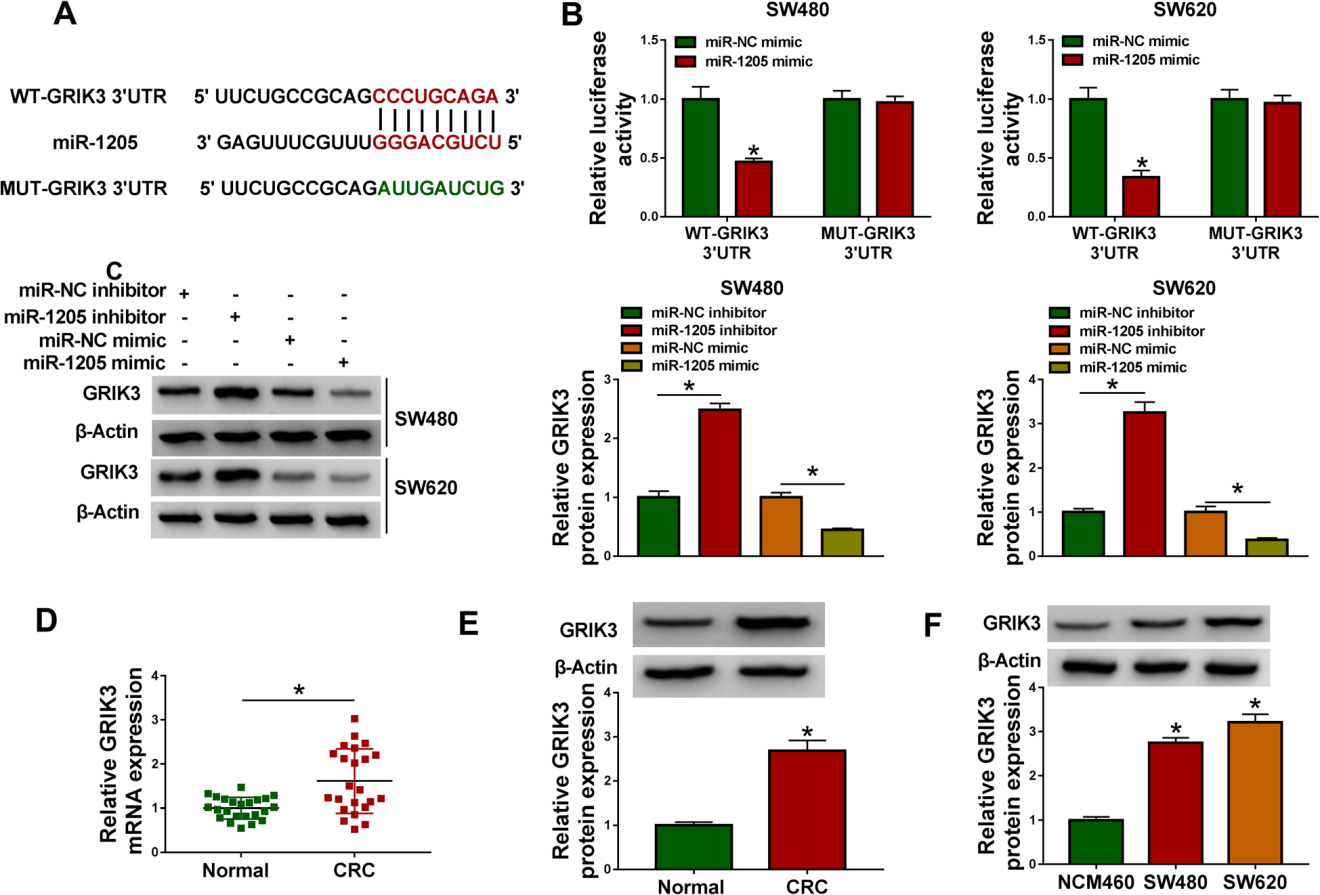
MiR-1205 bound to GRIK3 in CRC cells. **A** The binding sequence between miR-1205 and GRIK3 was assessed by miRDB online database. **B** The binding relationship between miR-1205 and GRIK3 was confirmed by dual-luciferase reporter assay. **C** MiR-1205 inhibitor upregulated GRIK3 protein expression, and miR-1205 mimic downregulated GRIK3 protein expression. **D**–**F** GRIK3 expression was upregulated in CRC tissues as well as SW480 and SW620 cells. Significant differences in (**B** and **E**) were compared with two-tailed Student’s *t* tests, in (**C** and **F**) with ANOVA and in (**D**) with Wilcoxon rank-sum test. *P < 0.05

Given the effects of miR-1205 inhibitor on CRC progression, the mRNA associated with miR-1205 was further sought. As a result, we found that there were 10 genes that were reported to regulate cancer progression and possessed the binding sequence of miR-1205. QRT-PCR then displayed that the expression of EGLN3, SOGA1, ARF6, and GRIK3 was apparently reduced by miR-1205 mimic, especially GRIK3 (Fig. S6). Thus, GRIK3 was regarded as a candidate. As shown in Fig. 5A, GRIK3 3′UTR possessed the 9 binding sites of miR-1205. And dual-luciferase reporter assay illustrated that luciferase activity in WT-GRIK3 3′UTR and miR-1205 mimic group was obviously repressed in SW480 and SW620 cells, but there was no apparent change in MUT-GRIK3 3′UTR and miR-1205 mimic group (Fig. 5B). In addition, GRIK3 protein expression was significantly upregulated by miR-1205 inhibitor and was obviously downregulated after transfection of miR-1205 mimic (Fig. 5C). Furthermore, GRIK3 expression was detected in CRC tissues and cells. Results presented that the mRNA and protein expression of GRIK3 were apparently upregulated in CRC tissues or SW480 and SW620 cells compared with control groups (Fig. 5D–F). Our findings demonstrated that miR-1205 interacted with GRIK3 in CRC cells.

### MiR-1205 inhibited CRC process via binding to GRIK3

**Fig. 6:**
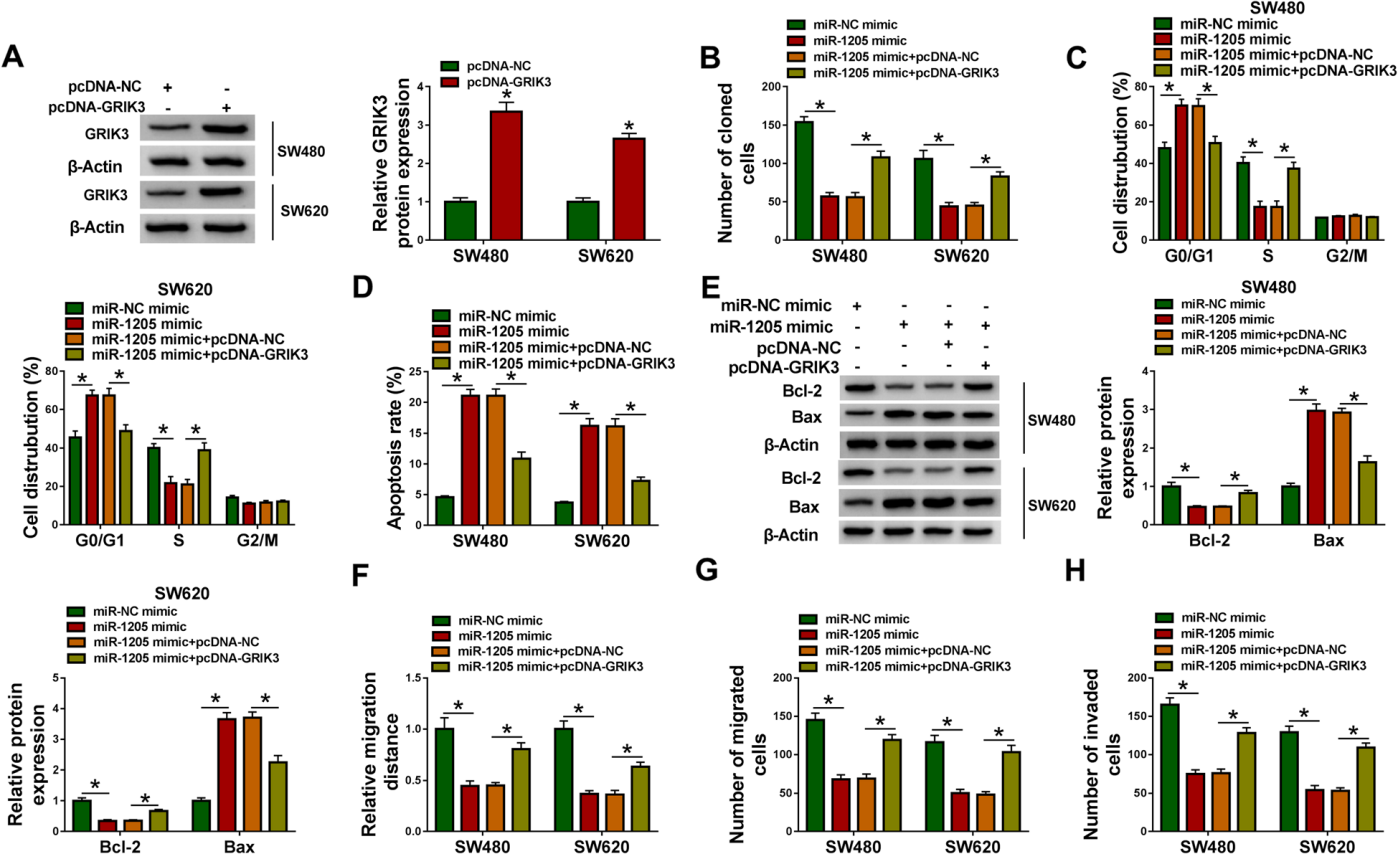
MiR-1205 mimic suppressed CRC development via interacting with GRIK3. **A** The high efficiency of pcDNA-GRIK3 in increasing GRIK3 protein expression was determined by western blot in SW480 and SW620 cells. **B** GRIK3 overexpression attenuated miR-1205-mediated repression on the colony-forming ability of SW480 and SW620 cells. **C**, **D** The effects of miR-1205 mimic on cell cycle process and cell apoptosis were hindered by ectopic GRIK3 expression. **E** The effects of miR-1205 on the protein expression of Bcl-2 and Bax were impaired by GRIK3 reintroduction. **F**–**H** MiR-1205-triggered inhibition on cell migration and invasion was reversed after GRIK3 overexpression. The *P* value in (**A**) were calculated with two-tailed Student’s *t* tests and in (**B**–**H**) with ANOVA. *P < 0.05

Whether miR-1205 regulated CRC progression by associating with GRIK3 was disclosed by rescue experiments. The overexpression efficiency of GRIK3 was firstly detected in SW480 and SW620 cells. The data from Western blot analysis showed that GRIK3 expression was dramatically increased in SW480 and SW620 cells transfected with pcDNA-GRIK3 (Fig. 6A), suggesting the high efficiency of pcDNA-GRIK3 in increasing GRIK3 expression. Subsequently, the data from Fig. 6B, C displayed that miR-1205 mimic repressed cell colony-forming ability and induced cell arrest in G0/G1 phase, whereas these effects were restored by GRIK3 overexpression. And miR-1205 was also disclosed to promote cell apoptosis; however, GRIK3 overexpression restrained this impact (Fig. 6D). Additionally, the influences between miR-1205 and GRIK3 on the expression of apoptosis-related proteins were unveiled. Results displayed that miR-1205 mimic distinctly downregulated Bcl-2 protein expression and upregulated Bax protein expression, but these impacts were relieved after GRIK3 overexpression (Fig. 6E). Furthermore, the migration and invasion of SW480 and SW620 cells were repressed by miR-1205 mimic, but enforced GRIK3 expression impaired these influences (Fig. 6F–H). These evidences implied that miR-1205 could regulate CRC progression through associating with GRIK3.

### CircASXL1 silencing downregulated GRIK3 expression by sponging miR-1205

**Fig. 7:**
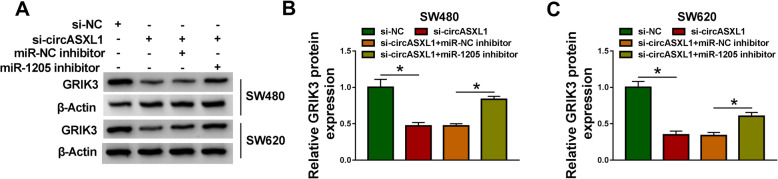
CircASXL1 regulated GRIK3 expression via binding to miR-1205. **A**–**C** CircASXL1 knockdown-mediated repression on the protein expression of GRIK3 was restrained by miR-1205 inhibitor in SW480 and SW620 cells. Significant differences were compared with ANOVA. *P < 0.05

It had been proved that circASXL1 was a sponge of miR-1205, and that miR-1205 was associated with GRIK3, whether circASXL1 regulated GRIK3 expression by interacting with miR-1205 was disclosed in this part. Western blot analysis showed that circASXL1 knockdown significantly downregulated GRIK3 protein expression, whereas miR-1205 inhibitor attenuated this effect (Fig. 7A–C). This evidence meant that circASXL1 controlled GRIK3 expression by interacting with miR-1205.

### CircASXL1 knockdown repressed tumor formation by regulating miR-1205/GRIK3 axis in vivo

**Fig. 8:**
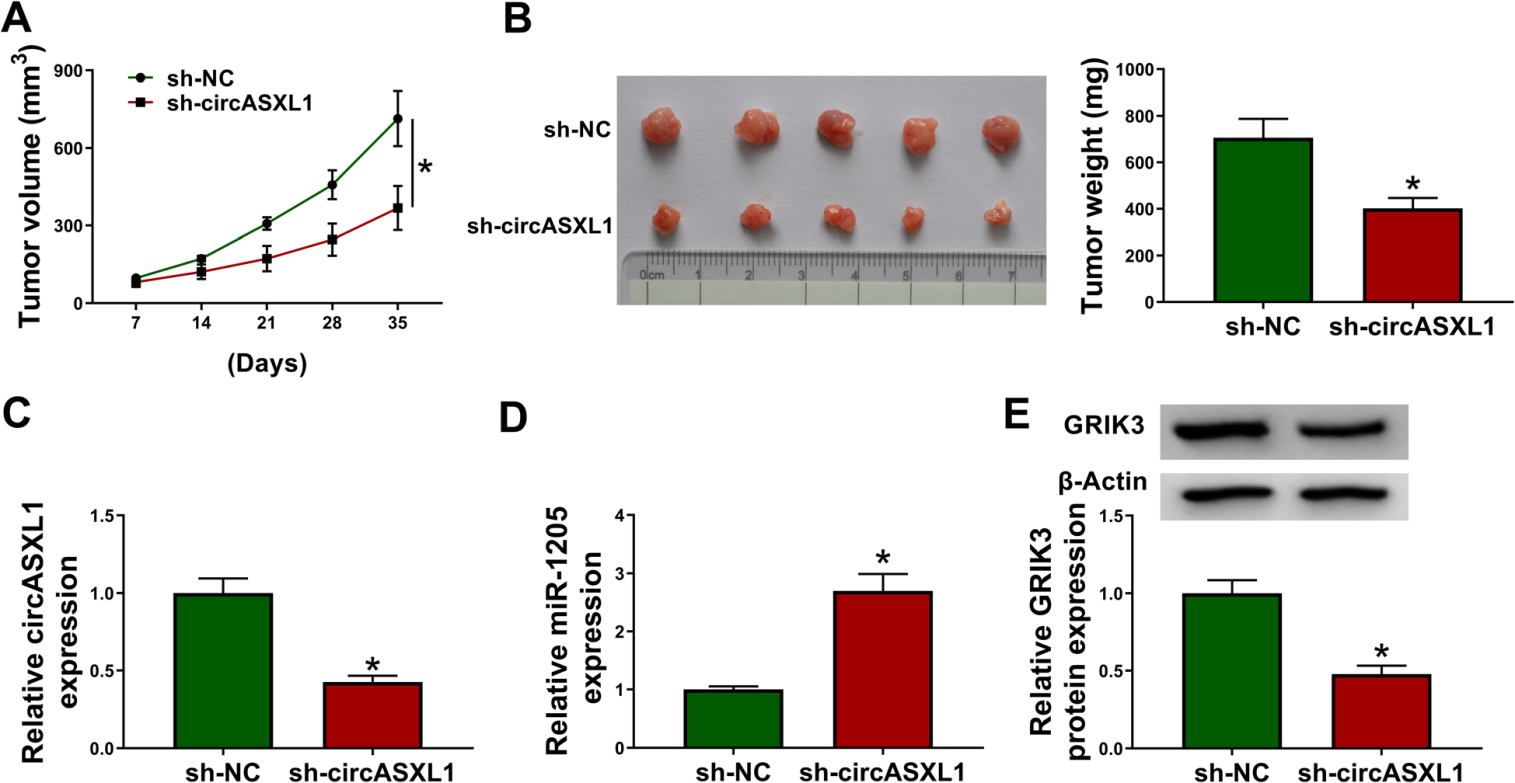
CircASXL1 knockdown inhibited tumor growth by upregulating miR-1205 expression and downregulating GRIK3 expression in vivo. **A**, **B** CircASXL1 knockdown reduced the volume and weight of tumors. **C**, **D** CircASXL1 knockdown decreased circASXL1 expression, and increased miR-1205 expression in vivo. **E** CircASXL1 silencing downregulated GRIK3 protein expression. Significant differences were compared with two-tailed Student’s *t* tests. *P < 0.05

To evidently demonstrate the role of circASXL1 in CRC progression, the effects of circASXL1 on tumor formation were further identified by in vivo assay. Results showed that circASXL1 silencing inhibited the volume and weight of forming tumors (Fig. 8A, B). Additionally, the impacts of circASXL1 knockdown on the expression of miR-1205 and GRIK3 were unveiled. QRT-PCR data firstly presented that circASXL1 was dramatically weakly expressed in sh-circASXL1 group than in sh-NC group (Fig. 8C). And results demonstrated that circASXL1 silencing obviously upregulated miR-1205 expression and downregulated GRIK3 protein expression in the neoplasms from sh-circASXL1 group (Fig. 8D, E). It was analyzed using IHC assay that the expression of GRIK3 and Ki67 were lower in sh-circASXL1 group than in sh-NC group (Fig. S7A–C). Therefore, all these data showed that circASXL1 absence repressed tumor growth via regulating miR-1205 and GRIK3 in vivo.

## Discussion

CircRNA exerts function by sponging miRNA, thereby regulating alternative splicing or mediating precursor gene expression [9, 32], by which it participates in regulating the development of many aggressive tumors, including CRC [33]. In recent years, multiple researchers have focused revealing the pathogenic mechanism of CRC through circRNA to improve the overall survival scores of CRC patients. For example, Shen et al. explained that circ_0026344 hindered tumor metastasis by sponging miR-183 [34]. Geng et al. also reported that circ_0009361 silencing contributed to cell proliferation, migration, and invasion via binding to miR-582 [35]. In another example, circRNA amyloid precursor-like protein 2 (circAPLP2) was indicated to facilitate cell proliferation and metastasis, and repress cell apoptosis [36]. In spite of many achievements made by researches in unveiling the genesis of CRC, the molecular mechanism behind circRNA regulating CRC development is still unclear. In this study, we found another mechanism that circASXL1 acted as an oncogene in CRC by modulating miR-1205/GRIK3 axis.

CircASXL1, located in chr20:30954186-30956926 and formed by exons 2 and 3 of ASXL1, has been revealed to have correlation with both tumor stage and lymph node invasion in bladder cancer [37]. In leukemia, the low expression of circASXL1 repressed the growth of THP-1 monocytes [38]. These data suggested the oncogenic role of circASXL1 in cancer malignant progression. In this paper, we found that circASXL1 expression was obviously upregulated in CRC tissues and cells. Loss-of-function experiments showed that circASXL1 absence hindered cell proliferation as well as migratory and invasive abilities, and accelerated cell apoptosis in CRC. Furthermore, it was demonstrated that circASXL1 silencing hindered tumor formation in vivo. These results also suggested that circASXL1 acted as a tumor promoter in CRC. As reported early, ASXL1, circASXL1 patient, has been reported to be negatively correlated with lymph node metastasis of CRC [38], and to inhibit CRC cell proliferation through acting as a target of miR-3187-3p [39], which was opposite to the function of circASXL1. Additionally, Li et al. revealed that exon–intron circRNAs, located in cell nucleus, modulated their parental transcript in a cis-acting way [39]. However, we reported that circASXL1 was an exonic circRNA. Thus, circASXL1 might not mediate ASXL1 expression. These evidences also manifested that circASXL1-mediated regulation on CRC progression might be not correlated with ASXL1.

Emerging evidences presented that circRNA could regulate cancer progression via sponging miRNA through serving as a competing endogenous RNA (ceRNA) [40, 41]. Thus, the circASXL1-associated miRNA(s) was further explored. As a result, we identified that circASXL1 interacted with miR-1205. According to the current data, miR-1205 played important roles in cancer progression. For example, Wang et al. reported that miR-1205 contributed to cell growth in prostate cancer [42]. Yang et al. also unveiled that circ_0034642 accelerated cell proliferative and invasive abilities by sponging miR-1205 in glioma [43], suggesting that miR-1205 functioned as a tumor suppressor. In this study, the repressing role of miR-1205 in CRC malignant progression was identified. It was found that miR-1205 was lowly expressed in CRC tissues and cells. Results showed that miR-1205 inhibitor hindered the inhibitory effects of circASXL1 silencing on the proliferative, migratory, and invasive capacities, and the promoting influence of that on cell apoptosis in CRC, implying that circASXL1 knockdown inhibited CRC progression through interacting with miR-1205 and that miR-1205 was a tumor suppressor in CRC process. In the study of Jiang et al., miR-1205 was found to repress epithelial-to-mesenchymal transition (EMT) in CRC cells [44], which supported the results that miR-1205 repressed the migration and invasion of CRC cells. Additionally, it was revealed that miR-1205 repressed the proliferation and invasion of gastric cancer cells through interaction with circRNA cytoplasmic FMR1 interacting protein 2 (circCYFIP2) [22]. Considering that both gastric cancer and CRC belong to digestive system-related diseases, circCYFIP2 may affect circASXL1-mediated CRC progression through competitively binding to miR-1205.

MiRNA can silence gene via repressing bound-mRNA expression, thereby modulating the progression of human diseases [40]. Therefore, the mRNA interacted with miR-1205 was sought. Through the screening using miR-1205 mimic, GRIK3 was employed as a target gene of miR-1205. Additionally, Du et al. also indicated that GRIK3 repressed CRC process by inhibiting cell proliferation and migration [45]. Therefore, we guessed that miR-1205 might regulate CRC process by binding to GRIK3. To this end, gain-of-function experiments were performed. Our data demonstrated that GRIK3 overexpression attenuated miR-1205 mimic-mediated action, which proved our hypothesis. Additionally, we found that GRIK3 expression was obviously upregulated in CRC tissues and cells. As we all know, there was no data on the cell cycle, invasion, and apoptosis of CRC cells mediated by GRIK3. And our study was the first one to demonstrate that GRIK3 promoted cell invasion and hindered cell cycle arrest and cell apoptosis in CRC.

## Conclusion

CircASXL1 functioned as an oncogene in CRC malignant progression by regulating cell proliferation, motility, and apoptosis. And the underlying mechanism was that circASXL1 worked as a ceRNA to sponge miR-1205, thereby inducing GRIK3 expression. Our findings provide a novel mechanism for revealing circRNA-mediated regulation of CRC. Importantly, the novel mechanism suggests that targeting circASXL1/miR-1205/GRIK3 pathway may be an effective therapeutic target for CRC.

## Supplementary Information

**Additional file 1: Figure S1.** The schematic diagram showed circASXL1 was formed from exons 2 and 3 of ASXL1 with 195 base pairs (bp) in size.

**Additional file 2: Figure S2.** The prediction of circASXL1 expression and the overall survival curve. (A) CircASXL1 was highly expressed in CRC tissues according to the prediction of GEO dataset (GSE142837). (B) CRC patients with high circASXL1 had poor prognosis. Significant differences were analyzed with Wilcoxon rank-sum test in (A) and log-rank test in (B). *P<0.05.

**Additional file 3: Figure S3.** CircASXL1 knockdown restrained CRC progression. (A) CircASXL1 silencing reduced circASXL1 expression in SW480 and SW620 cells. (B) The colony-forming ability of SW480 and SW620 cells was inhibited after circASXL1 knockdown. (C and D) CircASXL1 silencing induced cell arrest and apoptosis. (E and F) CircASXL1 depletion decreased Bcl-2 protein expression, and increased Bax protein expression. (G-I) Cell migration and invasion were repressed by circASXL1 absence. Significant differences were compared with two-tailed Student’s t-tests. *P<0.05.

**Additional file 4: Figure S4.** CircASXL1 depletion repressed the migration of SW480 and SW620 cells under mitomycin C treatment. Significant differences were compared with two-tailed Student’s t-tests. *P<0.05.

**Additional file 5: Figure S5.** CircASXL1 silencing upregulated the expression of miR-1205, miR-206 and miR-767-3p, but had no effects on the expression of miR-1-3p, miR-34b-5p and miR-616-3p. Significant differences were compared with two-tailed Student’s t-tests. *P<0.05.

**Additional file 6: Figure S6.** The expression of miR-1205-assocaited genes was detected by qRT-PCR in SW480 and SW620 cells transfected with miR-1205 mimic or miR-NC mimic. Significant differences were compared with two-tailed Student’s t-tests. *P<0.05.

**Additional file 7: Figure S7.** CircASXL1 silencing reduced the expression of GRIK3 and Ki67 in the neoplasms from in vivo tumor formation assay. *P<0.05.

**Additional file 8: Table S1.** The sequences of primers and oligonucleotides used in this study.

## Data Availability

The data sets used and/or analyzed during the current study are available from the corresponding author on reasonable request.
