# Supplementary figures and images for: CircASXL1 knockdown represses the progression of colorectal cancer by downregulating GRIK3 expression by sponging miR-1205
Source: World J Surg Oncol. 2021 Jun 14;19:176. doi: 10.1186/s12957-021-02275-6 (PMC8204566; doi:10.1186/s12957-021-02275-6)

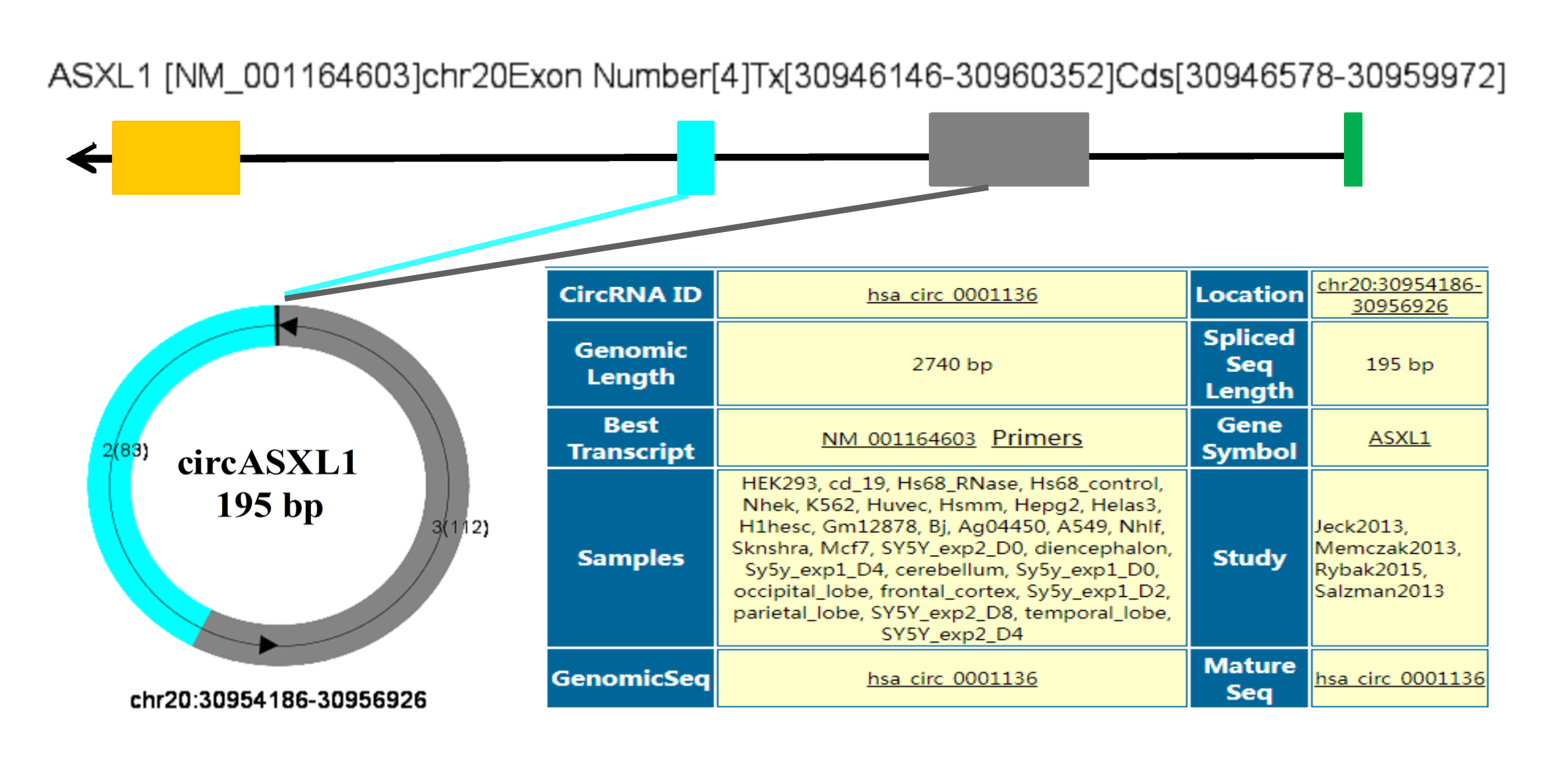

Supplement: Supplementary file 1 — Additional file 1: Figure S1. The schematic diagram showed circASXL1 was formed from exons 2 and 3 of ASXL1 with 195 base pairs (bp) in size. [file 12957_2021_2275_MOESM1_ESM.tif]

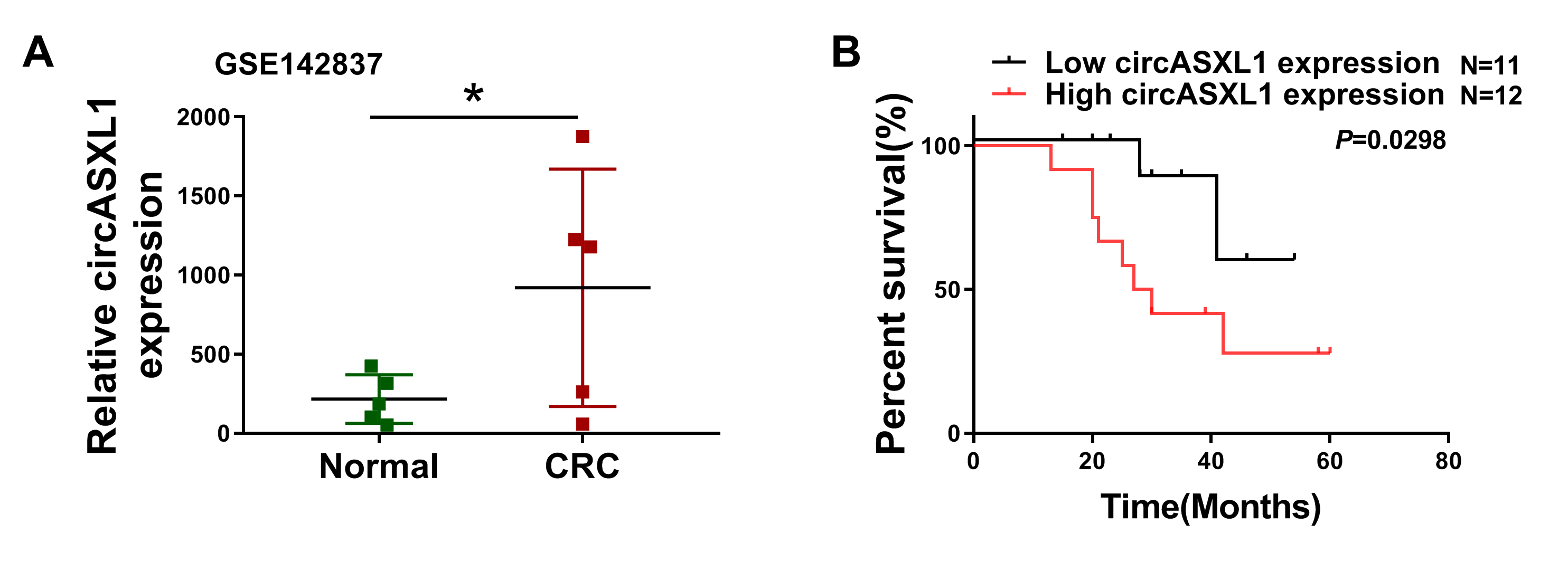

Supplement: Supplementary file 2 — Additional file 2: Figure S2. The prediction of circASXL1 expression and the overall survival curve. (A) CircASXL1 was highly expressed in CRC tissues according to the prediction of GEO dataset (GSE142837). (B) CRC patients with high circASXL1 had poor prognosis. Significant differences were analyzed with Wilcoxon rank-sum test in (A) and log-rank test in (B). *P<0.05. [file 12957_2021_2275_MOESM2_ESM.tif]

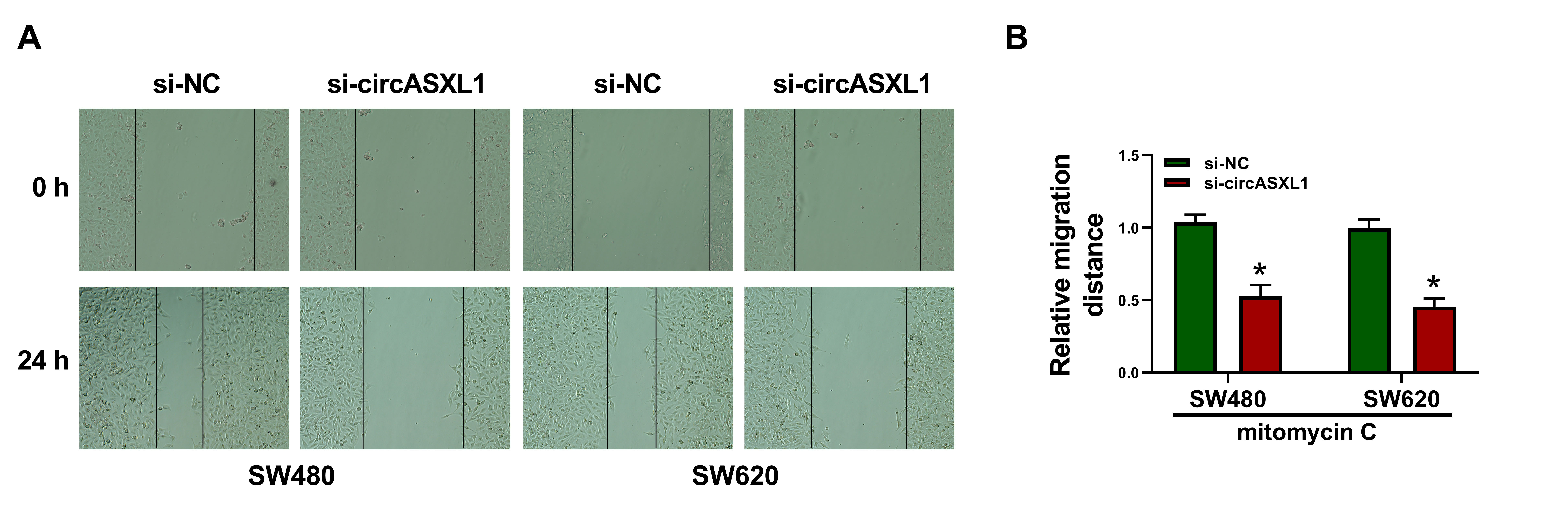

Supplement: Supplementary file 4 — Additional file 4: Figure S4. CircASXL1 depletion repressed the migration of SW480 and SW620 cells under mitomycin C treatment. Significant differences were compared with two-tailed Student’s t-tests. *P<0.05. [file 12957_2021_2275_MOESM4_ESM.jpg]

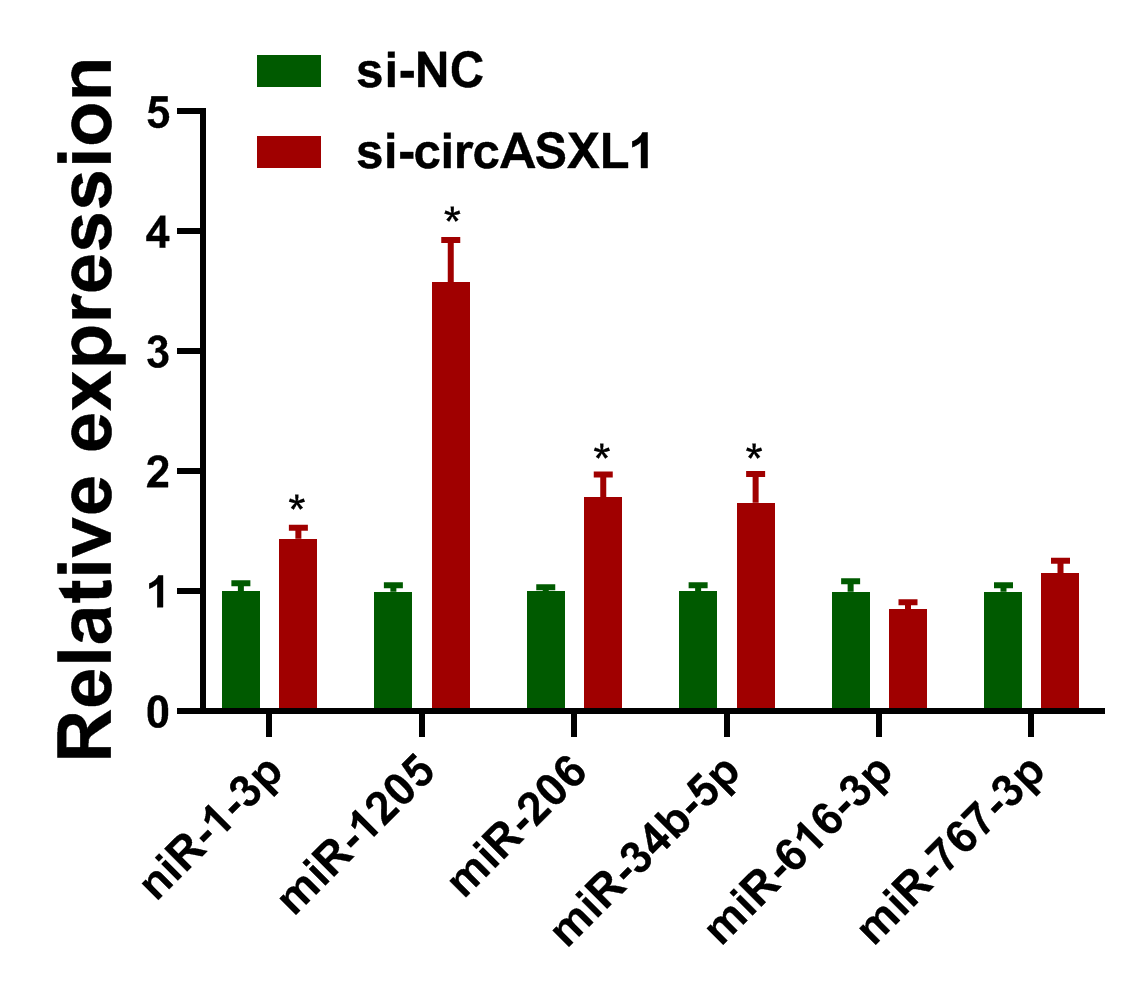

Supplement: Supplementary file 5 — Additional file 5: Figure S5. CircASXL1 silencing upregulated the expression of miR-1205, miR-206 and miR-767-3p, but had no effects on the expression of miR-1-3p, miR-34b-5p and miR-616-3p. Significant differences were compared with two-tailed Student’s t-tests. *P<0.05. [file 12957_2021_2275_MOESM5_ESM.tif]

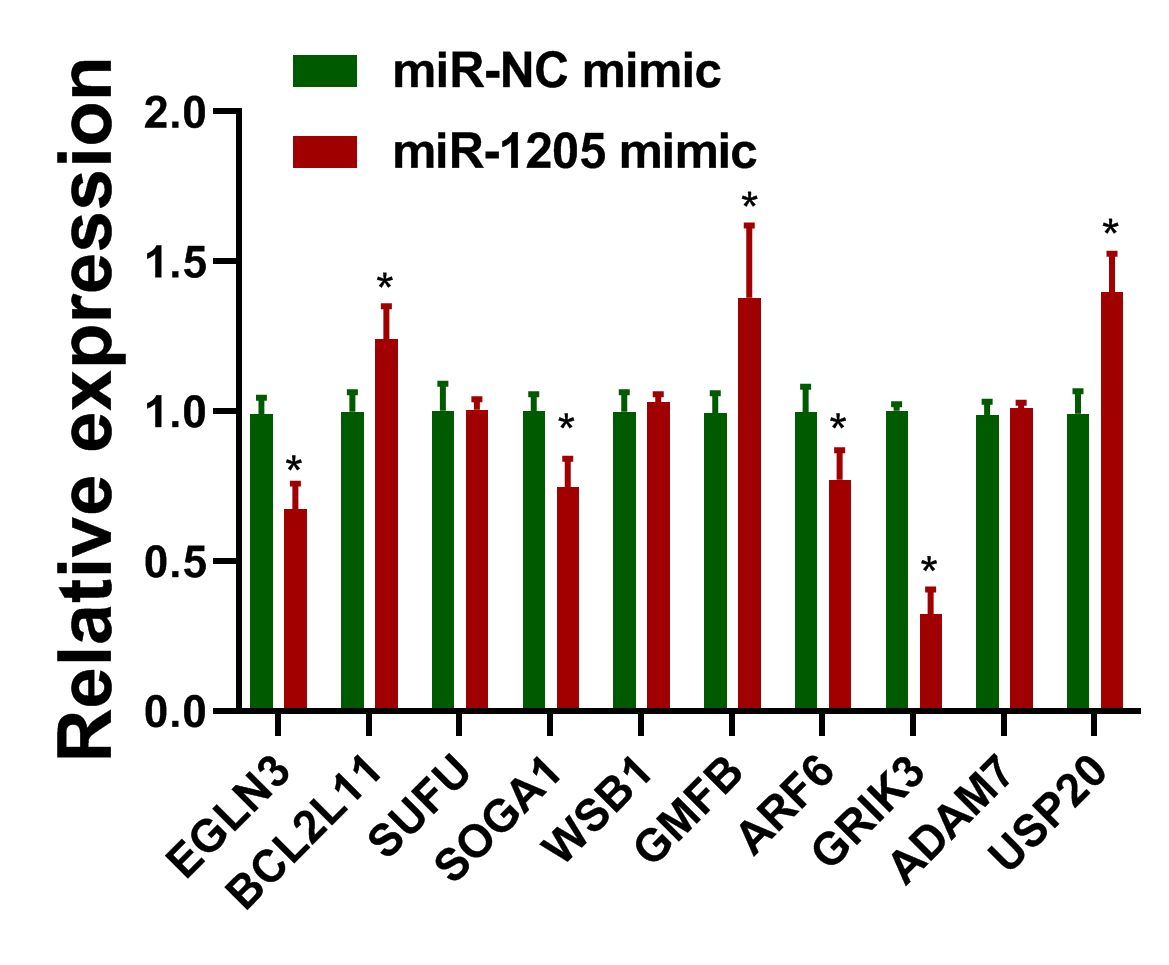

Supplement: Supplementary file 6 — Additional file 6: Figure S6. The expression of miR-1205-assocaited genes was detected by qRT-PCR in SW480 and SW620 cells transfected with miR-1205 mimic or miR-NC mimic. Significant differences were compared with two-tailed Student’s t-tests. *P<0.05. [file 12957_2021_2275_MOESM6_ESM.tif]

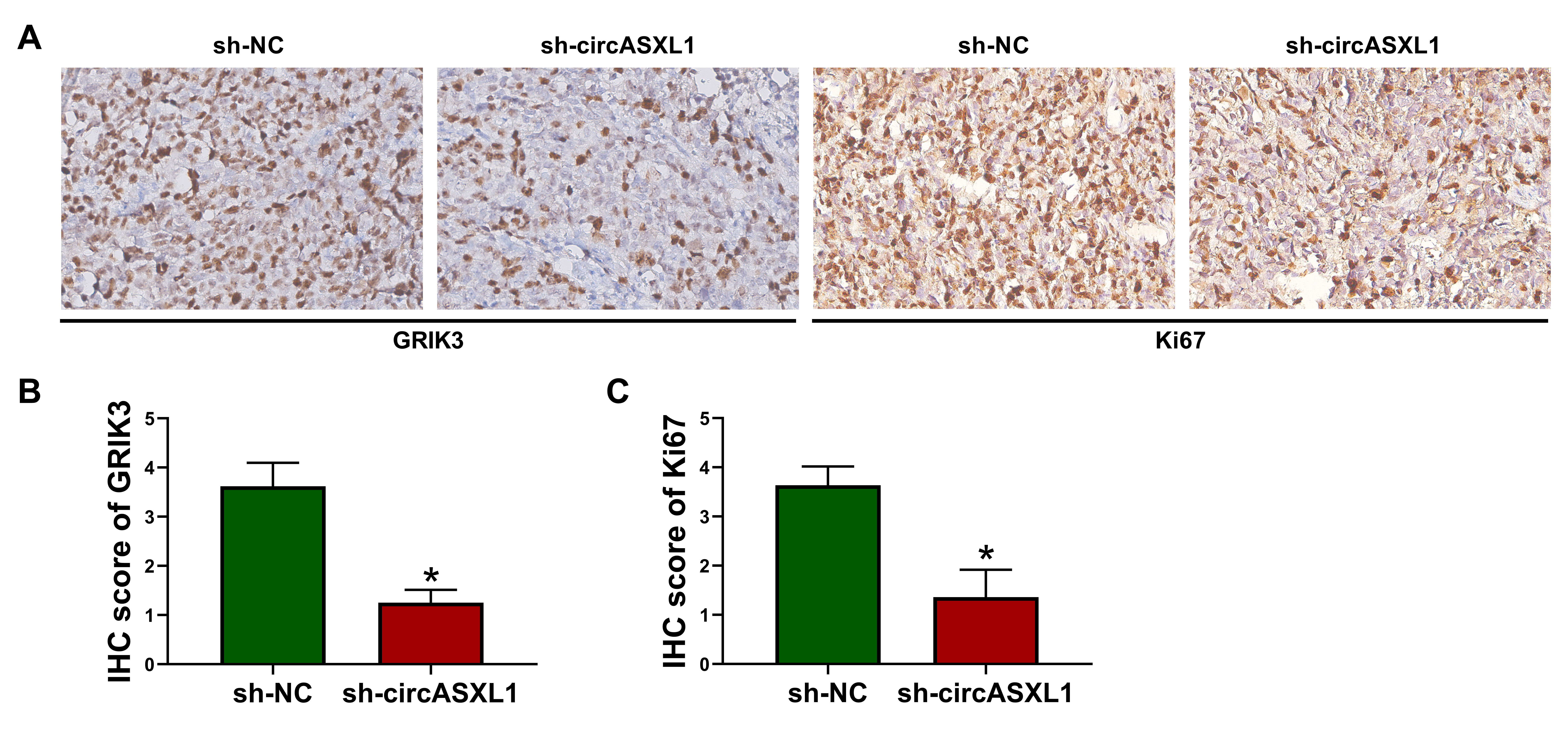

Supplement: Supplementary file 7 — Additional file 7: Figure S7. CircASXL1 silencing reduced the expression of GRIK3 and Ki67 in the neoplasms from in vivo tumor formation assay. *P<0.05. [file 12957_2021_2275_MOESM7_ESM.jpg]
