# Supplementary material for: CircASXL1 knockdown represses the progression of colorectal cancer by downregulating GRIK3 expression by sponging miR-1205
Source: World J Surg Oncol. 2021 Jun 14;19:176. doi: 10.1186/s12957-021-02275-6 (PMC8204566; doi:10.1186/s12957-021-02275-6)
Supplement: Supplementary file 8 — Additional file 8: Table S1. The sequences of primers and oligonucleotides used in this study. [file 12957_2021_2275_MOESM8_ESM.docx]

| Gene | Sequences of primers/oligonucleotides (From 5’ to 3’) |
| --- | --- |
| si-circASXL1 | GCTCAAGGTATTAGAAAACTA |
| si-circASXL1#2 | CACGCTCAAGGTATTAGAAAA |
| miR-1205 mimic | UCUGCAGGGUUUGCUUUGAG |
| miR-1205 inhibitor | CUCAAAGCAAACCCUGCAGA |
| si-NC | CCATTTACCCGAACGGCAA |
| miR-NC mimic | UUUGUACUACACAAAAGUACUG |
| miR-NC inhibitor | CAGUACUUUUGUGUAGUACAAA |
| circASXL1-F | ATGCCTCAATGCTATGCT |
| circASXL1-R | TTCTGCCTCTATGACCTG |
| ASXL1-F | TCGACGTGAGGGTGAAATGA |
| ASXL1-R | CAAGATGCGTTTGCTCGGTG |
| miR-1205-F | ACACTCCAGCTGGGTCTGCAGGGTTTGC |
| miR-1205-R | TGGTGTCGTGGAGTCG |
| GRIK3-F | GGCTCCATCTGTCTGCCTTT |
| GRIK3-R | TGCTCAGACCTGCGAAAGAG |
| GAPDH-F | GGTCACCAGGGCTGCTTT |
| GAPDH-R | GGAAGATGGTGATGGGATT |
| U6-F | CTCGCTTCGGCAGCACA |
| U6-R | AACGCTTCACGAATTTGCGT |
| miR-206-F | GCCGAGTGGAATGTAAGGAAGT |
| miR-206-R | TGGTGTCGTGGAGTCG |
| miR-34b-5p-F | GGGTAGGCAGTGTCATTAGC |
| miR-34b-5p-R | AACAACCAACACAACCCAAC |
| miR-616-3p-F | ACACTCCAGCTGGGAGTCATTGGAGGGTTT |
| miR-616-3p-R | TGGTGTCGTGGAGTCG |
| miR-767-3p-F | TCGGCAGGTCTGCTCATACCCCATG |
| miR-767-3p-R | TGGTGTCGTGGAGTCG |
| EGLN3-F | CCAAGCTACATGGTGGGA |
| Gene | **Sequences of primers/oligonucleotides (From 5’ to 3’)** |
| EGLN3-R | TCTGGTTGCGTAAGAGGG |
| BCL2L11-F | CACCAGCACCATAGAAGAA |
| BCL2L11-R | ATAAGGAGCAGGCACAGA |
| SUFU-F | CCGAGGATGACGAGGACA |
| SUFU-R | ATTCTGCCGCTGAGGGTT |
| SOGA1-F | AATGAGGACGCTGTTTGTG |
| SOGA1-R | TGGCTGCTCTGTCACTCG |
| WSB1-F | TACACTGCTCTTGGGTCG |
| WSB1-R | CAATGGTTTCAGGCTCGT |
| GMFB-F | TTGTGATGTTGCCGAAGA |
| GMFB-R | CAGTACCACCAGGCGTTT |
| ARF6-F | CGGCGACTGGAGAAATCA |
| ARF6-R | GCGAAACCGAAACGAAGC |
| ADAM7-F | CCTGGTTATTGTCGGTAT |
| ADAM7-R | TTGGCTCAGTCCTTATCT |
| USP20-F | GCGGAACGGAGTGAAGTAC |
| USP20-R | AGGAGACGTGGCTGTTGAT |

**Table S1. Primer sequences and oligonucleotide sequences used in the present research**
